# Murine 3T3-L1 Adipocyte Cell Differentiation Model: Validated Reference Genes for qPCR Gene Expression Analysis

**DOI:** 10.1371/journal.pone.0037517

**Published:** 2012-05-22

**Authors:** Tatjana Arsenijevic, Françoise Grégoire, Valérie Delforge, Christine Delporte, Jason Perret

**Affiliations:** 1 Laboratory of Pathophysiological and Nutritional Biochemistry, Université Libre de Bruxelles, Brussels, Belgium; 2 Nutrisub ASBL, Brussels, Belgium; Kyushu Institute of Technology, Japan

## Abstract

**Background:**

Analysis of gene expression at the mRNA level, using real-time quantitative reverse transcription polymerase chain reaction (qRT-PCR), mandatorily requires reference genes (RGs) as internal controls. However, increasing evidences have shown that RG expression may vary considerably under experimental conditions. We sought for an appropriate panel of RGs to be used in the 3T3-L1 cell line model during their terminal differentiation into adipocytes. To this end, the expression levels of a panel of seven widely used RG mRNAs were measured by qRT-PCR. The 7 RGs evaluated were ß-actin (ACTB), glyceraldehyde-3-phosphate dehydrogenase (GAPDH), hypoxanthine phosphoribosyl-transferase I (HPRT), ATP synthase H+ transporting mitochondrial F1 complex beta subunit (ATP-5b), tyrosine 3-monooxygenase/tryptophan 5- monooxygenase activation protein, zeta polypeptide (Ywhaz), Non-POU-domain containing octamer binding protein (NoNo), and large ribosomal protein L13a (RPL).

**Methodology/Principal Findings:**

Using three Excel applications, GeNorm, NormFinder and BestKeeper, we observed that the number and the stability of potential RGs vary significantly during differentiation of 3T3-L1 cells into adipocytes. mRNA expression analyses using qRT-PCR revealed that during the entire differentiation program, only NoNo expression is relatively stable. Moreover, the RG sets that were acceptably stable were different depending on the phase of the overall differentiation process (i.e. mitotic clonal expansion versus the terminal differentiation phase). RPL, ACTB, and Ywhaz, are suitable for terminal differentiation, whereas ATP-5b and HPRT, are suitable during mitotic clonal expansion.

**Conclusion:**

Our results demonstrate that special attention must be given to the choice of suitable RGs during the various well defined phases of adipogenesis to ensure accurate data analysis and that the use of several RGs is absolutely required. Consequently, our data show for the first time, that during mitotic clonal expansion, the most suitable RGs are ATP-5b, NoNo and HPRT, while during terminal differentiation the most suitable RGs are, NoNo, RPL, ACTB and Ywhaz.

## Introduction

Adipose tissue is essentially composed of adipocytes, wherein lipogenesis and lipolysis take place within the frame of energy storage and release, in response to the energy balance status. In addition to their key role in the control of energy metabolism, adipocytes are also considered as endocrine cells due to their secretion of adipokines, which are highly influential on, e.g. the immune system, blood vessels and insulin sensitivity [1]. When adipocytes develop excessively or inappropriately, they are considered as a risk factor that may lead to obesity, cardiovascular diseases, diabetes and cancer [2]. Consequently, signals affecting adipocyte differentiation and function are currently of considerable interest. Adipocyte growth arrest and differentiation require combinatorial signals involving extracellular environment and cues, intracellular and transcriptional effectors as well as unknown serum factors.

In the 3T3-L1 cell culture model, temporary exposure of the cells to a combination of insulin, glucocorticoid and an inducer of cAMP signaling triggers adipogenesis, changing the expression of hundreds of structural genes and a variety of transcription factors [3]. Contact inhibition arrests preadipocyte proliferation at confluence. Upon stimulation of differentiation, they first reenter the cell cycle, undergo several rounds of division called mitotic clonal expansion (MCE), and then undergo the terminal differentiation (TD) [3]. These events produce dramatic changes in the expression of a host of genes and proteins [4].

Real-time quantitative reverse transcription polymerase chain reaction (qRT-PCR) is a rapid and sensitive method for gene expression measurement. Given the low amounts of mRNA in fat cells, qRT-PCR became the method of choice for gene expression studies in adipocytes. Despite being a very powerful technique, qRT-PCR is an indirect method of measurement subjected to significant variability during the various stages throughout the experimental protocol (e.g. input sample, RNA extraction, efficiency of reverse transcription from RNA to complementary DNA and PCR efficiency). This may lead to misinterpretation of the results [5]–[7]. The accepted and validated method to deal with these difficulties is to normalize the transcript level of the gene of interest to a set of other genes commonly termed as the reference genes (RGs). However, it is essential that the expression of the RGs be stable, i.e. not be affected by the experimental conditions used in the study under investigation.

Nevertheless, there is substantial evidence now suggesting that the expression of so called internal RG often vary significantly under different experimental conditions. Adipocyte differentiation is a process where cells undergo enormous morphological changes, over a period of several days. It is accompanied by substantial biochemical changes such as cell cycle exit, changes in biochemical processes and metabolism and alteration in structural proteins [8]–[11]. Significant modifications of gene expression therefore underlie these vast arrays of protein and cellular changes. As commonly used RGs are mostly structural proteins or enzymes involved in metabolism, it is especially important to validate the stabilities of these genes during the process of adipocyte differentiation. Although many studies have investigated gene expression changes in 3T3-L1 cells, very few have evaluated the stability of the RGs used as being appropriate and reliable RGs for qPCR normalization in this model, i.e. compliant to the now widely accepted criteria originally described by Bustin, Vandesompele and Pfaffl [12]–[14].

The aim of our study was to identify and validate a set of appropriate normalizing RGs for the studies of 3T3-L1 cell differentiation into adipocytes, being the main and widely used *in vitro* model.

## Results

### Selection of Candidate Reference Genes

Seven candidates RGs were selected for this study. The RGs used were a standard panel of widely used and recognized RGs that are either part of commercially available RGs sets or have been described in the literature as such. Special attention was also given to select those RGs whose proteins belong to different functional classes, to reduce the chances of RGs being co-regulated. Due to the extensive use of β-actin (ACTB) and glyceraldehyde-3-phosphate dehydrogenase (GAPDH) as RG for gene expression during adipocyte differentiation, they were of course also included in our analysis (see Table 1 for full gene name accession number and function). For each RG, primer efficiency was calculated from a standard curve covering 4 logs, and was typically between 95 to 105%, in line with MIQE (minimum information for publication of quantitative real-time PCR experiments) guidelines [15], [16] (see Table 2 for primer sequences, amplicons sizes and efficiencies).

**Table 1:** Symbols, names, accession numbers and function of the candidate RGs evaluated.

Gene symbol	Gene name	Accession number	Function
**ACTB**	β-actin	NM_007393	Involved in cell motility, structure and integrity
**GAPDH**	Glyceraldehyde-3-phosphate dehydrogenase	NM_008084.2	Carbohydrate metabolism
**RPL 13a**	Ribosomal protein large L13a	NM_009438	Structural constituent of ribosome
**HPRT**	Hypoxanthine phosphoribosyl-transferase I	NM_013556	Purine synthesis through the purine salvage pathway
**ATP-5b**	ATP synthase, H+ transporting mitochondrial F1 complexbeta subunit	NM_01677	Subunit of mitochondrial ATP synthase
**Ywhaz**	Tyrosine 3-monooxygenase/tryptophan 5–monooxygenaseactivation protein, zeta polypeptide	NM_011740	Protein domain in specific binding
**NoNo**	Non-POU-domain containing, octamer binding protein	NM_023144	DNA- and RNA binding protein

**Table 2:** Gene symbols, primer sequences, amplicon sizes and primer efficiencies of the candidate RGs evaluated.

Gene symbol	Primers sequence (5′-3′)	Amplicon size (bp)	Primer efficiency
**ACTB**	F: CCTGTGCTGCTCACCGAGGC	174	0.984±0.004
	R: GACCCCGTCTCTCCGGAGTCCATC		
**GAPDH**	F: TGCACCACCAACTGCTTAGC	177	0.945±0.018
	R: GGATGCAGGGATGATGTTCT		
**RPL 13a**	F: GGCTGCCGAAGATGGCGGAG	131	0.972±0.006
	R: GCCTTCACAGCGTACGACCACC		
**HPRT**	F: ACATTGTGGCCCTCTGTGTG	162	0.981±0.013
	R: TTATGTCCCCCGTTGACTGA		
**ATP-5b**	F: TGGCTCAGAGGTGTCTGC	165	0.985±0.008
	R: TCAGTCAGGTCATCAGCAGG		
**Ywhaz**	F: AAAAACAGCTTTCGATGAAGCC	168	1.002±0.013
	R: GCCGGTTAATTTTCCCCTCC		
**NoNo**	F: TGCTCCTGTGCCACCTGGTACTC	170	1.001±0.020
	R: CCGGAGCTGGACGGTTGAATGC		

Primer efficiencies are expressed as the mean ± SEM of n  = 3 separate experiments.

### Analysis of RNA Purity and Integrity

RNA was extracted from 3T3-L1 cells at different stages of adipocytes differentiation as described in Materials and Methods. The 260/280 and 260/230 OD ratios were measured to respectively assess the purity of RNA samples with respect to protein contamination and to residual organic solvent. All RNA used showed a 260/280 and 260/230 OD ratios between 1.8 and 2.0, indicative of good RNA free from protein and organic solvent contaminations. RNA integrity was also assessed by Bioanalyzer analysis on an Experion automated electrophoresis system delivering RNA integrity verification and RNA concentration quantification. The algorithm assigns a RNA quality integrity number (RQI) score from 1 to 10, where 10 represents ideally intact RNA and 1 represents a highly degraded RNA. Experion analysis of all RNA samples yielded RQIs higher than 7 (Table 3), indicative of high quality RNA.

**Table 3:** Summary of RNA integrity analysis.

Sample name	RQI
D0	7.9
MCE 4 h	9.7
MCE 8 h	9.8
MCE 12 h	9.6
MCE 16 h	9.8
MCE 20 h	9.9
MCE 24 h	9.6
MCE 48 h	10.0
MCE 60 h	9.8
TD day 3	8.8
TD day 5	8.0
TD day 7	8.2
TD day 9	7.7

RNA integrity was assessed by Bioanalyzer analysis and the algorithm assigned a RQI score from 1 to 10, where 10 represents ideally intact RNA and 1 represents a highly degraded RNA.

### Expression Profiling of Candidate Reference Genes

**Figure 1:**
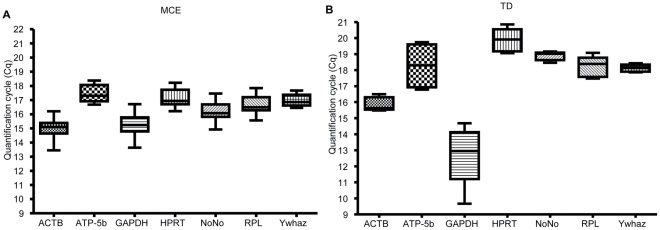
Distribution of qPCR quantification cycle values for the candidate RGs. qPCR gene expression analysis was carried out on cDNA derived from differentiating cells. Quantification cycles values (Cqs) were determined for the expression of ACTB, ATP-5b, GAPDH, HPRT, NoNo, RPL and Ywhaz candidate RGs during MCE (A) and TD (B). More highly expressed genes are associated with lower Cqs.

The expression of 7 candidates RGs was investigated during differentiation of 3T3-L1 cells into adipocytes using SYBR green based qPCR chemistry. The obtained quantification cycles (Cqs) were variable between candidate RGs, but more noteworthy also between the two differentiation phases, i.e. MCE and TD (Figure 1). The distribution of RG Cqs provides a global representation of the variation of RG expression as well as information on their relative abundance (Figure 1). More highly expressed genes are associated with lower Cqs. As expected in adipocytes, the expression of ACTB and GAPDH is high throughout the differentiation program, in MCE (Figure 1A) as well as during TD (Figure 1B). However, this did not imply that these two genes (i.e. ACTB and GAPDH) were stable throughout adipogenesis. Consequently, it was necessary to test the expression stability of other RG candidates.

### Stability of Reference Genes within Samples with Respect to Differentiation Phases

To examine the impact of the variability of RG expression throughout differentiation of adipocytes, the expression stability of 7 RGs was tested during the proliferation phases MCE and TD of adipocytes. RG expression levels were measured by qRT-PCR and their stabilities were evaluated by the three most widely used algorithms i.e. GeNorm [13], NormFinder [17] and BestKeeper [18]. The analysis was first conducted during the entire differentiation program, i.e. MCE and TD, taken together as one experiment as typically done. Only NoNo turned out to be sufficiently stable during the entire differentiation process of 3T3-L1 cells into adipocytes. However, no accepted minimal number of stable RGs (at least 3) to be used for gene expression studies could be identified by GeNorm. Indeed, as according to MIQE [16], one RG cannot be considered suitable for normalization. Therefore, we decided, based on the significant morphological, biochemical and gene expression shifts occurring between the MCE and TD phases, to consider and test MCE and TD phases as independent experiments with respect to the RG assayed using all three software algorithms evaluating RG stability.

**Figure 2:**
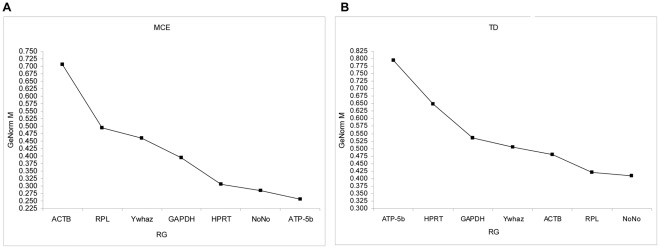
Expression stability values of candidate RGs during MCE and TD assessed by GeNorm. qPCR gene expression analysis was carried out on cDNA derived from differentiating cells. Gene expression data were converted to relative expression using the relationship (E+1)^−ΔCt^ and data was analyzed using GeNorm algorithm, yielding the rank order of gene expression stabilities during MCE (A) and TD (B). M, the average stability measure, was determined for each candidate RG. RGs with the lowest M are considered as most stable.

**Figure 3:**
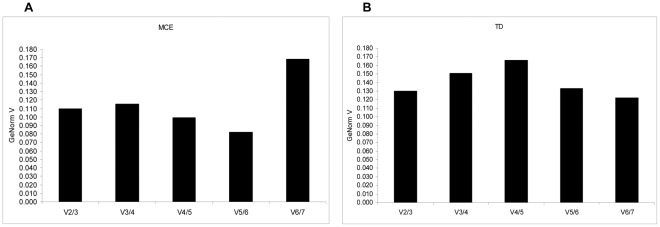
Determination of optimal number of RGs for qPCR data normalization by geNorm. Determination of the optimal number of RG for the normalization of qPCR data analysis was performed using the geNorm algorithm. The V parameter is used to determine the optimal number of RGs required normalizing the expression of candidate genes. the V cut-off value was set to 0.15, below which the inclusion of an additional RG is no longer required. V2/3 is based on the geometric mean of the normalization factors of ATP-5b, Nono and HPRT during MCE (A), and NoNo, RPL and ACTB during TD (B) of 3T3-L1 cells.

The GeNorm algorithm applies a statistical algorithm to calculate the average stability measure (M) of each candidate gene as the average pairwise variation (V) for that gene to the RGs. The RGs are then ranked by stepwise exclusion of the gene with the highest M. RGs with the lowest M are considered as most stable. Figure 2 shows the average M defined by GeNorm software package during MCE (Figure 2A) and TD (Figure 2B). Based on M values, the most stable genes, displaying the lowest M values, are ATP-5b, NoNo and HPRT during MCE (Fig 2A). Noticeably, of the two extensively used RGs (ACTB and GAPDH), ACTB offered the lowest stability throughout MCE. However, during TD, ACTB was amongst the four best ranking RGs along with NoNo, RPL, and Ywhaz (Figure 2B). The V parameter calculated by GeNorm is used to determine the optimal number of RGs required for normalizing the expression of genes of interest. The optimization of the number of RGs starts with the inclusion of the two genes with the lowest *M* value, and continues by sequentially adding genes with increasing values of *M*. Thus, GeNorm calculates the pairwise variation V*n*/V*n*_+1_ between two sequential normalization factors NF *n* and NF*n*_+1_ containing an increasing number of RG. A large variation means that the added gene has a significant effect on the normalization and should preferably be included for calculation of a reliable normalization factor [13]. As originally described by Vandesompele et al [13], the V cut-off value was set to 0.15, below which the inclusion of an additional RG is no longer required. In both MCE and TD, V 2/3 were respectively equal to 0.11 (Figure 3A) and 0.13 (Figure 3B), below the V cut-off value of 0.15.

These findings clearly illustrate the differences in stability of RGs in 3T3-L1 cells during the differentiation process. Furthermore, it shows for the first time as well that the set of RGs to be used varies according to the part of differentiation process under study (MCE or TD). During MCE, the best RG ranked according to M value was ATP-5b, that was however the least stable RG during TD. On the contrary, during TD, the GeNorm algorithm proposed to use ACTB as one of the best ranked RGs, which was on the other hand the least stable during MCE. These results indicate that neither one RG nor a unique set of RGs is appropriate for both MCE and TD. These data therefore stress the importance of using an appropriate set of RGs for each situation studied.

The RGs evaluation by BestKeeper and NormFinder tools are shown in tables 4 and 5. According to BestKeeper analysis, RGs with the lowest coefficient of variance (CV) represent the most stable RGs [18]. In this context, during MCE, our data showed ATP-5b as the most stable RG with a CV ± SD of 3.79±0.75. During TD, ACTB, NoNo, GAPDH and RPL were the four best ranking RGs with CV ± SD of 0.90±0.14, 1.19±0.23, 2.11±0.31 and 2.55±0.43, respectively.

**Table 4:** Analysis of RGs expression stability during MCE using BestKeeper and NormFinder algorithms.

MCE	ACTB	ATP-5b	GAPDH	HPRT	NoNo	RPL	Ywhaz
**n**	8	8	8	8	8	8	8
GM [Cq]*	15.46	19.90	15.46	15.46	15.46	15.46	15.46
AM [Cq]*	15.49	19.91	15.49	15.49	15.49	15.49	15.49
Min-Max [Cq]*	13.83–17.20	18.66–21.05	13.83–17.20	13.83–17.20	13.83–17.20	13.83–17.20	13.83–17.20
CV±SD*	5.29±0.82	3.79±0.75	5.29±0.82	5.29±0.82	5.29±0.82	5.29±0.82	5.29±0.82
**S**	0.796	0.194	0.266	0.232	0.307	0.311	0.141

n, number of samples; Cq, quantification cycle; GM, geometric mean; AM, arithmetic mean; Min Max: minimum and maximum; CV, coefficient of variance of RG; SD standard deviation; *BestKeeper statistics; S, stability value determined by NormFinder: expression stability decreases with increasing S value.

**Table 5:** Analysis of RGs expression stability during TD of adipocytes using BestKeeper and NormFinder algorithms.

TD	ACTB	ATP-5b	GAPDH	HPRT	NoNo	RPL	Ywhaz
**n**	5	5	5	5	5	5	5
GM [Cq]*	15.80	17.56	14.90	20.11	19.33	17.04	18.79
AM [Cq]*	15.80	17.59	14.90	20.13	19.34	17.05	18.81
Min-Max [Cq]*	15.60–16.03	16.30–18.76	14.61–15.49	18.54–21.22	19.06–19.66	16.50–17.61	17.78–19.84
CV ±SD*	0.90±0.14	5.22±0.92	2.11±0.31	3.78±0.76	1.19±0.23	2.55±0.43	2.78±0.52
**S**	0.094	0.636	0.367	0.539	0.464	0.350	0.361

n, number of samples; Cq, quantification cycle; GM, geometric mean; AM, arithmetic mean; Min Max: minimum and maximum; CV, coefficient of variance of RG; SD standard deviation; *BestKeeper statistics; S, stability value determined by NormFinder: expression stability decreases with increasing S value.

The NormFinder tool calculates a stability value (S) for the RGs. The lowest S value indicates the most stable RG expression [17]. According to NormFinder analysis, the best ranked RG for MCE is Ywhaz, followed by ATP-5b, while during TD it is ACTB that comes out as the best RG, followed by NoNo.

RG rankings obtained with all three algorithms (GeNorm, BestKeeper, and Normfinder) were compared for MCE (Table 6) and TD (Table 7). While RGs rankings are slightly different in the midfield positions, the top and bottom ranked genes are fairly constant. During MCE (Table 6) ATP-5b was top ranked by GeNorm and BestKeeper algorithms and second-top ranked by NormFinder algorithm, while ACTB was quoted as the least stable by all three algorithms. On the contrary, in TD (Table 7), ACTB was listed amongst the 3 most stable RGs by all three algorithms.

**Table 6:** Ranking of RGs stability during MCE of 3T3-L1 cells.

Rank	GeNorm	BestKeeper	NormFinder
1	ATP-5b	ATP-5b	Ywhaz
2	NoNo		ATP-5b
3	HPRT		HPRT
4	GAPDH		GAPDH
5	Ywhaz		NoNo
6	RPL		RPL
7	ACTB		ACTB

RG stabilities are ranked in order of decreasing expression stability.

**Table 7:** Ranking of RGs stability during TD of 3T3-L1 cells into adipocytes.

Rank	GeNorm	BestKeeper	NormFinder
1	NoNo	ACTB	ACTB
2	RPL	NoNo	NoNo
3	ACTB	GAPDH	Ywhaz
4	Ywhaz	RPL	GAPDH
5	GAPDH	Ywhaz	RPL
6	HPRT	HPRT	HPRT
7	ATP-5b	ATP-5b	ATP-5b

RG stabilities are ranked in order of decreasing expression stability.

## Discussion

Identifying optimal RGs for normalizing qRT-PCR based gene expression data during cellular differentiation has currently become a widely recognized problem [19], [20]. The proposed alternative for addressing this problem is to use a weighted average of several RGs [13], [21], [22]. Reviewing the literature, we were confronted with a wide choice of different RGs used to study adipocyte differentiation. Moreover, interpretation of the data relied usually only on one RG, rather than on a set of RGs. It has been clearly demonstrated that inappropriate RGs selection in adipocytes can have profound impact on data interpretation and study conclusions [23]. Therefore, the aim of our study was to identify and validate a potential set of appropriate RGs for normalizing qRT-PCR gene expression data in the 3T3-L1 cell model of differentiation into adipocytes *in vitro*. We focused on RGs already in use by groups involved in adipocyte biology research (such as NoNo, ACTB, GAPDH) and also added some other widely known RGs generally used as such, though seldom used in the adipocytes field (such as ATP-5b, HPRT, Ywhaz, and RPL).

To this end, we tested 7 candidate RGs using cDNA produced from 3T3-L1 cells at different stages of adipocytes differentiation. Gene expression levels of the 7 candidates RGs were measured by qRT-PCR and the expression stabilities were evaluated by three widely recognized algorithms that are GeNorm, NormFinder and BestKeeper.

We first evaluated the RGs panel throughout the entire 3T3-L1 adipocyte differentiating process. We found that NoNo is the best and only suited RG for normalization purposes during the entire adipocyte differentiation process. However, undeniably, the use of just one RG is now clearly recognized as a major pitfall leading to inappropriate data analysis methodologies resulting in the recurrent publication of data that are at best inconsistent and at worst irrelevant and even misleading [15], [24]–[27]. Indeed, as it is no longer acceptable to use one RG in normalization studies, a closer examination of our data lead us to propose the use of two separate panels of at least 3 reference genes for normalizing qPCR data during the two distinct phases of adipocyte differentiation: MCE and TD. Consequently, this work clearly reveals, for the first time, that two sets of RGs are needed: one for the MCE phase and another one for the TD phase and that none of those two sets of RGs can confidently cover gene expression analysis of the overall differentiation process.

Though ACTB was shown in our study to be rather stable during adipocytes TD, it was clearly not stable during the MCE phase, as assessed using the three different algorithms [24], [26]. In contrast, ATP-5b was shown to be stable during MCE, but unsuitable as RG during TD. As for ATP-5b, our results are in agreement with the study of Sugden et al. [28], where ATP-5b was shown to be a good RG for qRT-PCR studies in mouse fibroblasts**.** Therefore, we propose to use at least two separate sets of three RGs in the widely used *in vitro* model of 3T3-L1 cells differentiation into adipocytes, i.e. one set for MCE and one set for TD; to ensure proper data analysis for gene expression quantification.

Numerous papers reported the widespread use of GAPDH, ACTB and ribosomal RNA 18S (18s rRNA), expressed constitutively in adipose tissue, as single RG for qRT-PCR data normalization [29]–[31]. However, adipose tissue is a highly regulated and complex organ, and it has been shown that housekeeping genes can be regulated under hormonal stimulation or during adipogenic differentiation. For example, GAPDH expression was shown to be up-regulated by insulin during differentiation of brown adipocytes [32]. In the present study, we also showed that GAPDH is not a stable RG in both MCE and TD. Ferguson et al. showed that the use of ACTB as the only RG for qRT-PCR analysis of gene expression during adipocyte differentiation can lead to erroneous data interpretation [23]. Our data showed that ACTB is a stable RG during TD but not MCE. Consequently, a set of several RGs is necessary to lead to accurate data analysis and interpretation [15]. As for 18S rRNA, we did not include it in our study for several reasons, albeit it is being used as a suitable RG for adipose tissue by several groups [31]. First of all, the regulation of rRNA synthesis is carried out by RNA polymerase I, thus independently from synthesis of mRNA which is carried out by RNA polymerase II [33]. There is, as well, considerable imbalance between mRNA and rRNA fractions [34]. Moreover, using 18S, that does not contain any “intron like” sequences, renders it error prone if complete removal of contaminating genomic DNA in the RNA samples is not assured in the workflow. But moreover, 18S shows resistance to degradation as compared to mRNA [21] and therefore will not reflect the overall sample status with respect to the various steps involved in sample preparation and conversion to cDNA, the final amplification target. Finally, it has been shown that even 18S RNA transcription can underlie gene transcription changes [34].

In conclusion, based on the evidence provided in the present study, we propose that NoNo, which, has been already described as suitable reference gene for gene expression studies in human adipose tissue [35], is the most suited RG for normalization purposes throughout the entire differentiation program of 3T3-L1 cells. But, one RG can no longer be accepted for reliable gene expression data analysis. Consequently, it is strongly recommended to use several RGs that, taken together, offer the required overall stability. Furthermore, the novelty of the present study resides in the strong and original recommendation of using different sets of RGs in gene expression studies during the two distinct adipogenesis differentiation phases: whereby ATP-5b, NoNo and HPRT are the best suited RGs for MCE, while ACTB, NoNo, RPL and Ywhaz genes are the best suited RGs during TD.

## Materials and Methods

### Materials

Dulbecco’s modified Eagle’s medium (DMEM, 4.5 g/l glucose), streptomycin/penicillin, fetal bovine serum, horse serum and calf serum were obtained from Invitrogen (Carlsbad, CA, USA). Bovine serum albumin, bovine insulin, 3-isobutyl-1-methylxanthine (IBMX), and dexamethasone were purchased from Sigma (St. Louis, MO, USA).

### Cell Culture

3T3-L1 cells, a kind gift of Dr I. Pirson [36], were grown in DMEM supplemented with 10% calf serum, 100 U/ml penicillin and 100 mg/ml streptomycin in 8% CO_2_/humidified atmosphere at 37°C. Differentiation to adipocytes was induced 2 days post-confluence by incubating the cells in DMEM supplemented with 10% fetal bovine serum, 500 µM IBMX, 0.25 µM dexamethasone and 10 µg/ml insulin (XDI cocktail) for 60 h, and then maintained in the same medium without IBMX and dexamethasone. Cells were harvested at different time points: at day 0 (undifferentiated confluent cells), during MCE, and during TD up to day 9. During MCE, cells were harvested successively every 4 hours during the first 24 h following the induction of differentiation and then every 24 h until day 3, when TD had begun. Thereafter cells were harvested every 2 days. For RNA isolation and purification, cells were harvested by scrapping in 1 ml acid phenol/guanidinium thiocyanate solution (Purezol, Bio-Rad Laboratories, Hercules, CA, USA), and stored at −20°C until RNA preparation.

### RNA Isolation

RNA isolation was carried out using the Aurum Total RNA Fatty and Fibrous Tissue kit (Bio-Rad Laboratories, Hercules, CA, USA) according to the manufacturer’s instructions, with some minor modifications. Briefly, the relative volume of chloroform was increased by 50% to increase lipid solubility; an extra wash step was included and finally we doubled the amount of DNAse on column to ensure total and reproducible sample to sample residual genomic DNA removal. RNA concentration and purity were determined by A260, A280 and A230 optical density (OD) measurements and A260/A280 and A260/A230 ( = 1.7–2.0) ratio determination using a NanoDrop 8000 Spectrophotometer (Thermo Scientific, Schwerte, Germany). Furthermore, RNA integrity was assessed by bioanalyzer assay on an Experion automated electrophoresis system (Bio-Rad Laboratories, Hercules, CA, USA).

### Primer Design

Primers were designed using NCBI RefSeq sequence entry data that was submitted to the Primer-Blast program (NCBI Tools). Special attention was given to primer length, annealing temperature, base composition and 3′-end stability. To ensure the optimal DNA polymerization efficiency and amplification specificity, amplicon length was set between 100 bp to 180 bp. Primers were mandatorily separated, when possible, by at least one intron to minimize amplification from any contaminating genomic DNA that may remain after the RNA purification procedure.

### cDNA Synthesis and Real-time PCR

Total RNA was reversed transcribed from 1 µg of total RNA in a final reaction volume of 20 µl, using the High Capacity RNA-to-cDNA Kit (Applied Biosystems, Carlsbad, CA, U.S.A.), according to manufacture’s instructions.

The absence of potentially contaminating genomic DNA was determined in all samples using a protocol developed and validated in house. Briefly, 2 to 5 ng total purified RNA was directly submitted to a qPCR reaction using a control set of primers that yield no signal in the presence of genomic DNA and primers that amplify genomic DNA in all cases. Only those samples that were negative for both target genes used were considered suitable for use in our qPCR studies. We have shown that determination directly on the RNA preparation, for residual genomic contamination, is more sensitive than the so called classical RT minus reaction. Indeed, the reagents contained in the cDNA reaction buffer inhibit the overall amplification reaction, thereby decreasing the sensitivity of the evaluation.

### Quantitative Real-time PCR Procedure

The qPCR reaction setup and plate preparation were standardized and carried out according to standard operating protocols in our lab. The final reaction volume of 20 µl contained 10 µl of the 2x Sybergreen master mix from Eurogentec (WOUB MESA BLUE qPCR Master Mix Pl.SYBR Low Rox, Eurogentec, Seraing, Belgium), 5 µl of the forward and reverse primer mix (final concentration 250 nM per primer) and 5 µl of the target material. When cDNA samples were used as the amplification target the quantity per 20 µl reaction was 2 ng. Thermocycling was carried out in a StepOne Plus qPCR machine (Applied Biosystems, Carlsbad, CA, USA). After an initial polymerase activation step at 95°C for 5 minutes, amplification followed by 40 cycles of [95°C 15 sec –60°C 10 sec –72°C 20 sec with data acquisition at this stage] and the reaction finished by the built in melt curve.

### Analysis of Gene Expression Stability

Gene expression stability analysis and matching statistics were performed using three widely recognized RG normalization algorithms: GeNorm, NormFinder and BestKeeper [13], [14], [17]. Detailed description of the three applications can be found elsewhere [37].
